# Case Report: Successful treatment of mixed-phenotype acute leukemia with cauda equina syndrome as the initial manifestation

**DOI:** 10.3389/fonc.2026.1859413

**Published:** 2026-09-16

**Authors:** Xin Xu, Pei Zhang, Jianmin Zhang, Lei Zheng, Yuting Wang, Ping Wang, Yaqun Ding, Ya Tan, Shuangnian Xu, Ling Wei

**Affiliations:** 1 Department of Hematology, the First Affiliated Hospital of Army Medical University, Chongqing, China; 2 Department of Nuclear Medicine, the First Affiliated Hospital of Army Medical University, Chongqing, China; 3 Department of Radiology, the First Affiliated Hospital of Army Medical University, Chongqing, China

**Keywords:** allogeneic hematopoietic stem cell transplantation, cauda equina syndrome, mesenchymal stem cells, mixed phenotype acute leukemia, third-party umbilical cord blood stem cells

## Abstract

**Background:**

Mixed-phenotype acute leukemia (MPAL) has a poor prognosis, and initial presentation with cauda equina syndrome (CES) is extremely rare. Allogeneic hematopoietic stem cell transplantation (allo-HSCT) may be the only potentially curative modality. To our knowledge, this is the first reported case of MPAL presenting with CES at disease onset.

**Methods:**

We reviewed the clinical data of a 21-year-old man with high-risk myeloid/B-lymphoid MPAL presenting with CES as the initial manifestation. The patient initially presented with lumbosacral radicular pain and lower limb numbness, which rapidly progressed to fecal and urinary incontinence and inability to ambulate. Comprehensive imaging examinations and sacral biopsy ultimately confirmed myeloid/pre-B lymphoblastic MPAL with cauda equina and lumbosacral nerve root involvement. After the patient achieved complete remission with the combination chemotherapy regimen (MTRix + VD + BCL-2i), which features high blood–nerve-barrier penetration, the patient received myeloablative conditioning based on fractionated total-body irradiation and then underwent matched unrelated donor allo-HSCT. Third-party umbilical cord blood stem cells (UCBs) and mesenchymal stem cells (MSCs) were administered as adjunctive support. Graft-versus-host disease (GVHD) prophylaxis consisted of cyclosporine A, mycophenolate mofetil, and short-course methotrexate.

**Results:**

Neutrophil and platelet engraftment occurred on days +12 and +10 post-transplant, respectively. At the 24-month follow-up, the patient remained in minimal residual disease (MRD)-negative remission of the primary disease, free of acute and chronic GVHD, with motor strength recovery from grade 0 to grade 3.

**Conclusion:**

In conclusion, evidence-based multi-agent chemotherapy followed by allo-HSCT with third-party UCBs and MSC support resulted in favorable engraftment kinetics, adequate GVHD prophylaxis, and long-term remission. These outcomes support its clinical application. These findings highlight the promising efficacy and safety of this combined treatment approach in this high-risk MPAL case.

## 1 Introduction

Mixed-phenotype acute leukemia (MPAL) represents a highly heterogeneous malignant hematologic malignancy defined by the co-expression of myeloid and lymphoid lineage markers. Accounting for 1%–4% of adult acute leukemia (AL), MPAL is associated with low chemotherapy-induced remission rates, high relapse risk, and dismal prognosis (1–3). Allogeneic hematopoietic stem cell transplantation (allo-HSCT) remains the only potentially curative therapeutic option for these patients (4, 5).

Cauda equina syndrome (CES) results from compression, infiltration, or injury of the cauda equina nerve roots within the lumbosacral spinal canal and manifests with lumbosacral radicular pain, lower limb sensory-motor deficits, and fecal–urinary incontinence (6). Orthopedic and neurological pathologies, including lumbar disc herniation and primary spinal tumors, represent its most frequent etiologies. CES as an initial presentation of leukemic extramedullary infiltration is exceedingly rare: fewer than 20 case reports of leukemia-associated CES have been documented (7), and MPAL with CES has not been reported. These patients typically first present to orthopedics or rehabilitation departments, leading to delayed diagnosis and often irreversible neurological damage by the time a correct diagnosis is established. Furthermore, the blood–nerve barrier (BNB) around the cauda equina limits the penetration of chemotherapeutic drugs (8). Consequently, conventional chemotherapy generally fails to eradicate such lesions, conferring substantial relapse risk. To date, no standardized treatment regimen is available for this clinical entity.

The combination of classic chemotherapy regimens with targeted agents represents a new direction in leukemia therapy. Specifically, regimens combining BCL-2 inhibitors with chemotherapy achieve high response rates in MPAL (9). Total body irradiation (TBI) is a classic myeloablative conditioning regimen, uniquely effective in eradicating residual extramedullary lesions within chemotherapy-poor sanctuary sites. Fractionated TBI (fTBI) preserves antileukemic efficacy while reducing radiotherapy-related toxicity (10). Allo-HSCT supported by third-party umbilical cord blood stem cells (UCBs) and mesenchymal stem cells (MSCs) can overcome the limitations of unrelated donor transplantation—such as delayed hematopoietic reconstitution and high-incidence graft-versus-host disease (GVHD)—through synergistic interactions among these multicellular products, while potentiating the graft-versus-leukemia (GVL) effect (11–13).

To our knowledge, this is the first reported case of MPAL presenting with CES at initial diagnosis. Following complete remission achieved with a combination chemotherapy regimen exhibiting high BNB penetration, the patient received TBI-based conditioning and allo-HSCT supported by UCBs and MSCs, ultimately achieving sustained remission of the primary disease. This therapeutic strategy offers insights for the management of comparable clinical scenarios.

## 2 Case description

### 2.1 Patient’s history and physical examination

A 21-year-old man presented with lumbosacral radiating pain accompanied by right lower limb sensory abnormalities for 2 weeks, before rapidly progressing to fecal and urinary incontinence and inability to ambulate. Neurological examination revealed flaccid paraplegia, saddle-area sensory loss, and absent anal sphincter tone. The patient had an unremarkable past medical history, with no chronic comorbidities (e.g., hypertension and diabetes mellitus), no history of tobacco or alcohol use, no drug allergies, no history of lumbar trauma or lumbar disc herniation, and no relevant personal or family medical history.

### 2.2 Diagnostic workup

#### 2.2.1 Imaging examinations

Initial whole-body positron emission tomography–computed tomography (PET/CT) identified widespread hypermetabolic lesions involving the sacral canal, spinal canal, paravertebral soft tissues, pleura, and multiple lymph-node groups. These imaging findings were highly consistent with extensive leukemic dissemination (**Figure 1A**). Spinal MRI showed diffuse enhancement of the cauda equina and leptomeninges (**Figure 1C**).

**Figure 1:**
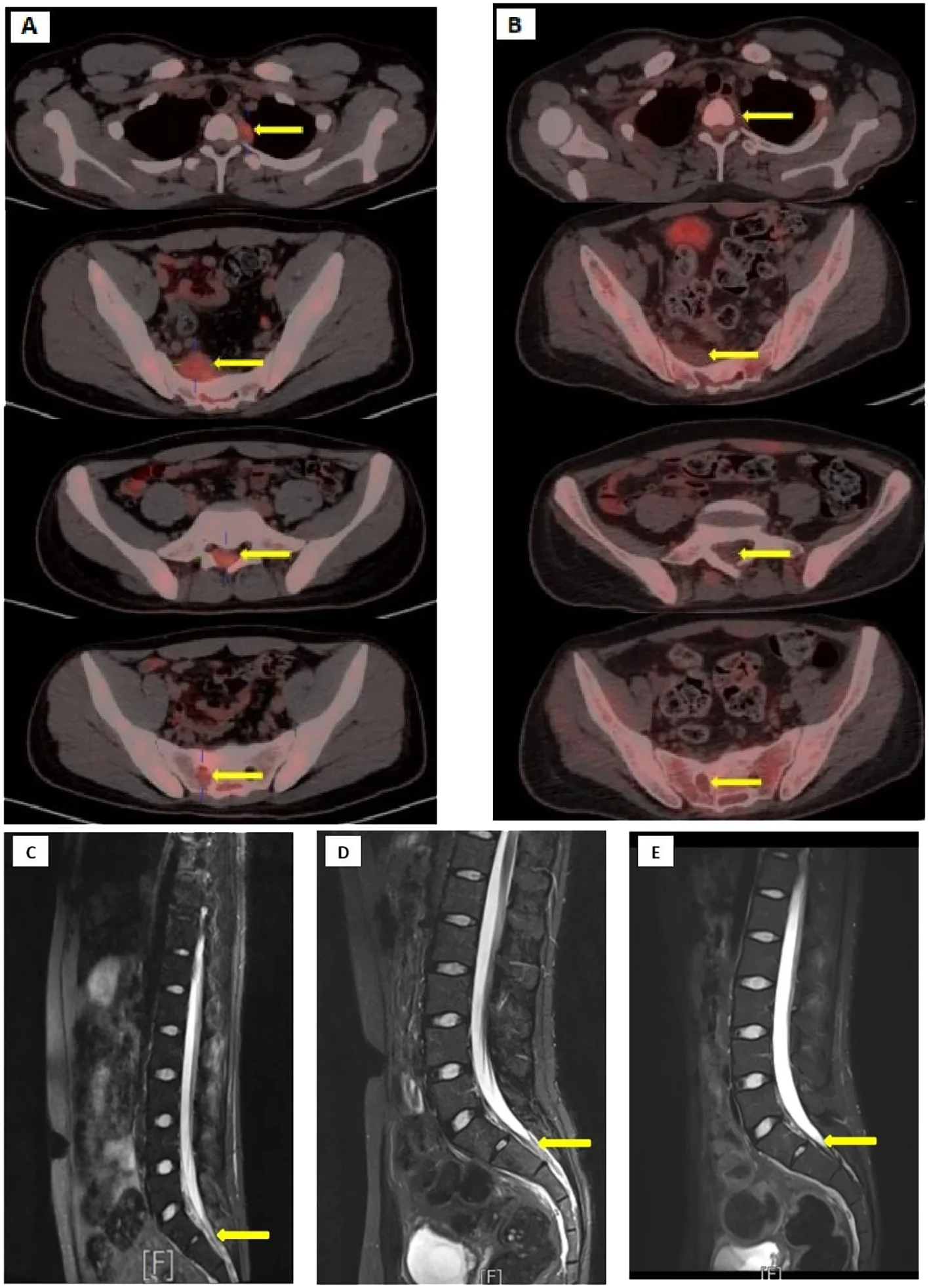
Diagnostic and post-treatment PET/CT and MRI. **(A)** Images from whole-body PET/CT at diagnosis: extensive, disseminated hypermetabolic disease (arrows) involving sacral/spinal canal, pleura, and lymph nodes. **(B)** Images from whole-body PET/CT at post-chemotherapy: suppressed metabolic activity of widespread disseminated lesions, with decreased size of presacral, right sacral foramen, intrasacral, and chest wall lesions compared with baseline. Complete resolution of intraspinal nodules, right obturator pelvic wall nodule, and left iliacus muscle nodule was observed. **(C, D)** Sagittal T1-weighted MRI of the lumbosacral spine at diagnosis and post-chemotherapy: diffuse, nodular leptomeningeal enhancement along the cauda equina nerve roots (arrowheads). **(E)** Sagittal T1-weighted MRI at 6 months post-transplant: the previously abnormal caudal enhancement has further diminished compared to the pre-transplantation state.

#### 2.2.2 Histopathology and laboratory examinations

Histopathological evaluation of sacral-canal biopsy demonstrated diffuse infiltration by blast cells. Staining was positive for CD20 (40%+), PAX-5 (60%+), CD5 (20%+), CD3 (20%+), Bcl-2 (+), C-myc (50%+), Ki-67 (70%+), CD43 (+), TdT (60%+), MPO (>90%+), CD117 (80%+), CD99 (50%+), CD34 (+), CD79a (70%+), and EBER (−); Bcl-6, CD10, MUM1, CD30, CD15, and CD4 were negative. Bone marrow MICM classification: Morphological (M) examination showed AL with 70% blasts; mast cells exhibited focal, scattered aggregates. Immunophenotyping (I) revealed that blasts accounted for 58.14% of nucleated cells and expressed CD117, CD33, CD38, CD13dim, CD45, HLA-DR, CD71, MPOdim, and CD56. Partial expression of CD34, CD19, and CD123 was detected, whereas CD5, CD7, CD2, CD4, CD16, CD10, CD11b, CD65, CD9, CD15, CD66c, CD36, CD64, CD14, CD300e, CD11c, CD105, and CXCR4 were absent. Cytogenetic (C) analysis showed 46, XY, t (8, 21) (q22; q22) [16]/47, idem, +der (21) t (8, 21) [4]. Molecular (M) genetic testing revealed an FLT3-ITD mutation (28% allelic ratio), a WT1 ratio of 9.1%, and a c-KIT D816V mutation; NPM1, IDH1, and IDH2 were negative. Bone marrow trephine biopsy exhibited sheet-like blast infiltration and grade 0 myelofibrosis. Immunophenotypes were scored per the 1998 European Group for the Immunological Characterization of Leukemias (EGIL) criteria, with a myeloid score of 3 and a B-lymphoid score of 2.5. Collectively, these results confirmed the diagnosis of myeloid/B-lymphoid MPAL (14).

#### 2.2.3 Diagnostic basis

At initial presentation, the patient complained of lumbosacral radicular pain, bilateral lower limb hypoesthesia, grade 0 motor strength, and combined anorectal–urinary sphincter dysfunction. Bladder impairment corresponded to a lower motor neuron-type (flaccid) neurogenic bladder, presenting with urinary retention and overflow incontinence. Combined with imaging findings, the diagnosis of complete CES was established according to the American Association of Neurological Surgeons (AANS) criteria (15). After excluding alternative etiologies, including lumbar disc herniation (MRI shows no disc herniation or nerve root compression, and intervertebral spaces remained intact), primary intraspinal neurogenic tumors (imaging demonstrated diffuse nerve root thickening and homogeneous enhancement rather than a sharply demarcated encapsulated mass), infectious diseases (the patient was afebrile, with normal white blood cell count and negative C‑reactive protein (CRP) and erythrocyte sedimentation rate (ESR)), and thalassemia-associated extramedullary hematopoiesis (no prior history of anemia, short and rapidly progressive disease course, absence of hepatosplenomegaly or other features of extramedullary hematopoiesis, and histopathology suggestive of MPAL), we suspected extramedullary infiltration by AL based on imaging and pathological findings.

### 2.3 Treatment response and clinical course

The patient received the IA regimen (idarubicin, 10 mg/m² daily on days 1–3; cytarabine, 150 mg/m² daily on days 1–7) as induction chemotherapy at a local hospital, and subsequent bone marrow examination demonstrated no remission (NR). The FLAG regimen (fludarabine 30 mg/m², cytarabine 2 g/m², and granulocyte‑colony stimulating factor (G-CSF) 5 μg/kg, all administered on days 1–5) was then given as re-induction chemotherapy. Bone marrow morphology showed complete remission; however, neurological symptoms remained unchanged. After admission to our department, the patient received an individualized, central nervous system (CNS)-directed intensive regimen (**Figure 2A**). PET/CT demonstrated a substantial extramedullary disease response (**Figure 1B**), whereas spinal MRI revealed no significant improvement of cauda equina lesions (**Figure 1D**). Consolidation therapy consisted of CLAG (**Figure 2B**) and intermediate-dose cytarabine (2 g/m² daily on days 1–7) combined with venetoclax and avapritinib (**Figure 2C**). Cerebrospinal fluid cytology and flow cytometry were performed during each cycle, alongside lumbar puncture with intrathecal chemotherapy for CNS leukemia prophylaxis (five times before transplantation). Post-transplant, serial lumbar punctures with intrathecal chemotherapy were continued until either primary disease relapse or 18 months post-transplant (15 total procedures). In addition, avapritinib maintenance was administered from post-transplant day +45 to month +24. This agent possesses favorable blood–brain barrier penetration, which may confer additional activity against CNS leukemia. All cerebrospinal fluid samples yielded negative results. The intrathecal regimen consisted of cytarabine 50 mg, methotrexate 10 mg, and dexamethasone 5 mg. High-risk disease features, together with cauda equina and lumbosacral nerve root involvement, rendered allo-HSCT necessary. A 10/10 HLA-matched, ABO-identical unrelated donor from the China Marrow Donor Program was identified and confirmed eligible for donation after workup. Myeloablative conditioning was delivered as follows: fTBI (single 5-Gy fraction daily for 2 days), cytarabine (3 g/m², day −6), etoposide (30 mg/kg, days −5 to −4), anti-thymocyte globulin (10 mg/kg, days −5 to −2), and cyclophosphamide (1.8 g/m², days −3 to −2). Stem cell infusion on day 0 included G-CSF-mobilized peripheral blood stem cells from the unrelated donor (mononuclear cell (MNC) 8.17 × 10^8^/kg, CD34^-^ 3.96 × 10^6^/kg), 4/6 HLA-matched third-party UCBs, and UCB-derived MSCs. GVHD prophylaxis consisted of cyclosporine A, mycophenolate mofetil, and short-course methotrexate. Based on baseline genetic profiles, avapritinib was combined with pre-transplant chemotherapy, and sorafenib was added for maintenance on post-transplant day +45. This combination resulted in grade 4 thrombocytopenia associated with mucocutaneous bleeding; platelet counts recovered after drug discontinuation, and thrombocytopenia recurred upon rechallenge. No further FLT3-targeted inhibitor therapy was subsequently administered. Concomitant supportive measures included antiemetic treatment with palonosetron hydrochloride plus aprepitant, antiviral prophylaxis with acyclovir, antifungal prophylaxis with posaconazole, transfusion support for hemoglobin <70 g/L or platelet count <20 × 10^9^/L, meropenem plus vancomycin anti-infective therapy for febrile neutropenia, and hematopoietic growth factor support with G-CSF and thrombopoietin. Major clinical events throughout the treatment course are summarized chronologically in **Table 1**.

**Figure 2:**
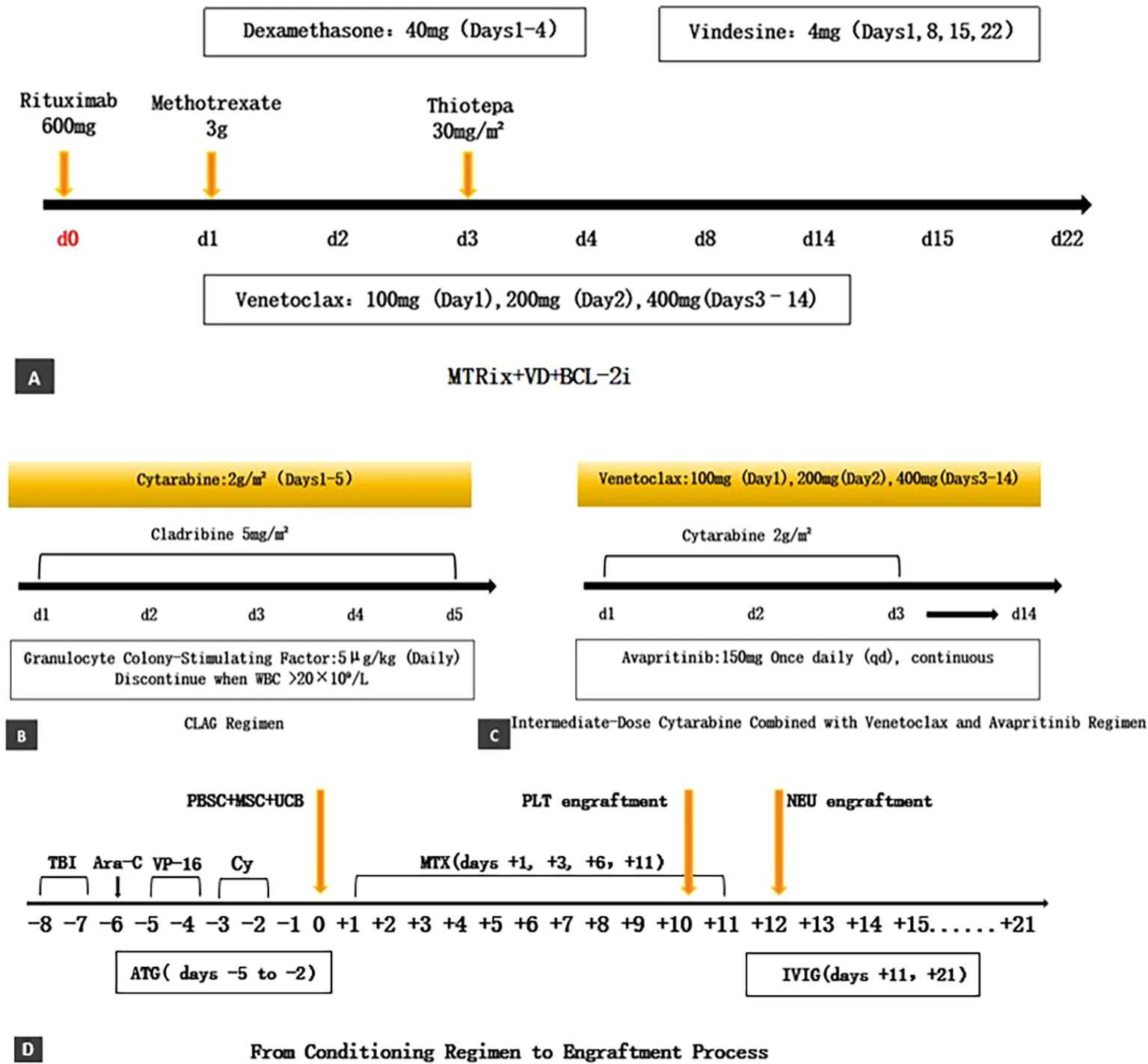
Therapeutic protocol. **(A)** MTRix + VD + BCL-2i regimen. MTRix: rituximab 375 mg/m^2^ on day 0, methotrexate 3 g/day on day 1, and thiotepa 30 mg/m² IV on day 3. VD: vincristine 4 mg/day on days 1, 8, 15, and 22; dexamethasone 40 mg/day on days 1–4. BCL-2i: venetoclax 100 mg PO on day 1, 200 mg on day 2, and 400 mg on days 3–14. **(B)** CLAG regimen: cladribine 5 mg/m², cytarabine 2 g/m², and G-CSF 5 μg/kg, all administered on days 1–5 (**C**). Intermediate-dose cytarabine with venetoclax and avapritinib: cytarabine 2 g/m²/day on days 1–3, avapritinib 150 mg qd continuous, venetoclax 100 mg PO on day 1, 200 mg on day 2, and 400 mg on days 3–14. **(D)** From conditioning regimen to engraftment process.

**Table 1:** Timeline.

Time	Clinical event/actions	Key findings	Outcome
Late March 2023	Lumbosacral radiating pain with right lower limb sensory abnormality (lasting for 2 weeks)	N/A	N/A
Late March to early May 2023	• Rapid progression: fecal and urinary incontinence and inability to ambulate • Neurological examination: flaccid paraplegia, saddle anesthesia, and absent anal sphincter tone	• Bone marrow aspirate + flow cytometry + cytogenetics + molecular genetics → diagnosed mixed-phenotype acute leukemia (MPAL) (myeloid/B lymphoblastic) • Imaging suggested extensive extramedullary/cauda equina nerve root involvement	Bedridden
Early May to late June 2023	Induction: IA “3 + 7” and FLAG regimens	Bone marrow assessment revealed no remission (NR) following initial chemotherapy	Bone marrow: CR Neurological symptoms: no change
Early July 2023	Transferred to our hospital	Bone marrow: CR CNS (−)	N/A
July–December 2023	Intensified regimen: R + MTRix + VD + BCL-2i (rituximab, methotrexate, thiotepa, vincristine, dexamethasone, and venetoclax) Consolidation: CLAG; IDAC + BCL-2i + avapritinib × 2 December 2023: urinary tract infection	Post-treatment PET/CT: marked suppression of systemic disseminated lesions (**Figures 1A,B**) MRI: persistent enhancement of the cauda equina nerve roots (**Figures 1C,D**) Bone marrow: CR CNS (−)	Spinal MRI still showed cauda equina enhancement (sanctuary persistence)
January–February 2024	Preparation: 10/10 HLA-matched unrelated donor selected (China Marrow Donor Program and ABO-identical) Conditioning regimen: fTBI + cytarabine + etoposide + BuCy (**Figure 2D**) GVHD prophylaxis: ATG + CsA + MMF + MTX	Day +2, fever, temperature 38.2 °C; day +31, nausea, vomiting, and anorexia; all symptoms resolved following symptomatic management	Platelet engraftment (day +10), neutrophil engraftment (day +12), and no GVHD or severe infection
February 7, 2024 (day 0)	Stem cell infusion: 1) G-CSF-mobilized PBSC from unrelated donor (MNC 8.17 × 10^8^/kg, CD34 3.96 × 10^6^/kg) 2) Third-party UCBs (4/6 HLA match) 3) Umbilical cord blood-derived MSCs	N/A	No infusion reactions or allergic reactions
March 24, 2024 (45 days post-transplant)	Bone marrow achieved complete remission, with negative MRD and undetectable fusion gene transcript	Maintenance therapy with sorafenib combined with avapritinib	Grade IV thrombocytopenia with mucocutaneous bleeding
August 2024 (6 months post-transplant)	Follow-up: clinical, laboratory, imaging, and chimerism (Pei Zhang)	Previously diffuse cauda equina root enhancement further diminished compared with pre-transplant (**Figure 1E**)	Objective cauda equina root radiologic improvement
February 2026 (24 months post-transplant)	Last follow-up: clinical, laboratory, imaging, and chimerism (Pei Zhang)	• Sustained morphological CR and MRD-negative • Full donor chimerism • No acute or chronic GVHD • Lower limb muscle strength improved from grade 0 to grade 3; partial activities of daily living regained	Patient perspective: significant clinical improvement, although lower limb strength not fully normalized

CR, complete remission; fTBI, fractionated total body irradiation; GVHD, graft-versus-host disease; UCBs, umbilical cord blood

Bone marrow examinations were performed at +1, +2, +3, +6, +9, +12, +18, and +24 months post-transplant, including morphology, flow cytometry, and FLT3-ITD mutation testing. Next-generation sequencing (NGS)-based gene mutation analysis was performed at +1, +3, +6, +12, and +24 months post-transplant. Routine hematological laboratory tests were obtained monthly. Follow-up data and relevant medical record collection were performed by Pei Zhang.

### 2.4 Outcomes

Neutrophil and platelet engraftment were achieved on day +12 and day +10, respectively. At the 24-month follow-up, no evidence of acute or chronic GVHD was observed throughout the surveillance period. At all post-transplant time points, bone marrow morphology and flow cytometry demonstrated blast counts within normal limits. FLT3-ITD, KIT D816V, and WT1 were undetectable. However, NGS repeatedly identified a DNMT3A mutation (variant allele frequency 0.8%–1.2%). The patient maintained deep remission [characterized by sustained complete morphological remission, minimal residual disease (MRD) negativity, and full donor chimerism]. MRI demonstrated substantial resolution of prior leptomeningeal and cauda equina enhancements (**Figure 1E**). Notably, the patient’s lower limb motor strength improved from grade 0 to grade 3 following transplantation.

Observed treatment-related adverse events included grade 2 oral mucositis, grade 2 vomiting, grade 4 neutropenia, and grade 4 thrombocytopenia. The patient developed only low-grade fever during the neutropenic phase; the fever resolved after symptomatic antipyretic and anti-infective therapy. No other serious adverse events were documented. All adverse events were evaluated and graded strictly according to the Common Terminology Criteria for Adverse Events version 5.0 (CTCAE v5.0).

## 3 Discussion

AL presenting initially with CES is extremely rare. Our PubMed literature review identified 11 previously reported cases: 9 acute myeloid leukemia (AML) cases, 1 acute lymphoblastic leukemia case (ALL), and 1 chronic myeloid leukemia (CML) case, including 3 post-transplant CNS relapses. With the exception of the case reported by Spiegelmann et al. (16)—an AML-M4 patient with CES secondary to extramedullary compression who achieved remission—all remaining patients died of disease relapse or infection, indicating a dismal prognosis. Relevant clinical details are summarized in **Table 2** (7, 16–24). To our knowledge, this is the first documented case of MPAL manifesting with this clinical phenotype. Such patients frequently lack typical leukemic symptoms in the early phase, and their disease is often misdiagnosed as compressive pathology, thus losing the opportunity for neurological functional recovery. Even after definitive diagnosis, the protective barrier property of the BNB poses substantial therapeutic challenges, and most patients suffer permanent neurological deficits (7, 20, 25). This underscores the need to include hematological malignancies within the differential diagnosis of rapidly progressive polyradiculopathies.

**Table 2:** Cases of acute leukemia complicated with cauda equina syndrome.

Case	Age/Sex	Diagnosis	Transplant	Treatment	Outcome
Petursson et al. (1981) (17)	21/M	Ph1+ AML	Yes	DA regimen + sacral radiotherapy; BMT	Died of interstitial pneumonia
Spiegelmann et al. (1988) (16)	35/M	AML-M5	No	Surgical resection of epidural mass + systemic chemotherapy + local radiotherapy	BM CR at 6 months, complete neurological recovery
Sandhu et al. (1998) (18)	22/M	Granulocytic Sarcoma	No	Systemic chemotherapy + radiotherapy	Relapsed and died at 4 months
Dalton et al. (2001) (19)	65/M	CML	Yes	Not documented	Died of relapse
Onal et al. (2006) (20)	41/F	AML-M4	Yes	systemic chemotherapy (Mitoxantrone plus cytarabine) + craniospinal irradiation+	No remission, lost to follow-up after discharge against medical advice.
Buakhao (2011) (21)	22/M	AML	No	Idarubicin + cytarabine + local radiotherapy	Died of infection
Verra et al. (2009) (22)	45/M	AML-M4	Yes	Laminectomy + radiotherapy + HD-Ara-C + DLI; intrathecal injection (IT) Ara-C	Died of relapse
Walton et al. (2020) (7)	72/M	AML	No	Comfort measures	Died
Li et al. (2022) (23)	20/M	B-ALL	No	Not detailed	Not detailed
Burns et al. (2025) (24)	60/M	AML	No	IT MTX + Ara-C + hydrocortisone twice weekly	No remission, lost to follow-up
Ali et al. (2026)	44/M	APL	No	FLAG-IDA + venetoclax induction → ATRA + ATO	The tumor was markedly shrunk, continuous follow-up
Our case	21/M	MPAL	Yes	systemic chemotherapy + HSCT	Survival with deep remission of the primary disease

It is widely accepted that achieving deep remission of primary disease prior to transplantation improves clinical prognosis. Currently, no standardized chemotherapy regimen exists for MPAL; available evidence is derived from retrospective cohorts, single-center series, and small-sample clinical trials. Meta-analyses suggest that ALL-like regimens yield higher remission rates and superior survival outcomes compared with AML-like regimens (26, 27), and combination regimens containing venetoclax confer substantial response rates in MPAL (9). Nevertheless, novel AML-like or hybrid regimens (e.g., CLAG/FLAG + M and MAED) also produce encouraging remission rates (28, 29). Individual case reports (30, 31) have described low-intensity chemotherapy combined with targeted or immunotherapeutic agents that achieved MRD-negative remission among elderly patients or individuals unfit for intensive chemotherapy. However, large-scale prospective studies remain absent. Induction therapy for MPAL is still investigational, and treatment regimens are tailored at each center according to individual patient features. Given the patient’s clinical features of widespread extramedullary infiltration and cauda equina involvement, we chose a modified “MTRix + VD + BCL-2i” regimen augmented with thiotepa and venetoclax (32)—agents with blood–brain barrier penetrance—to eliminate leukemic cells within sanctuary sites. Venetoclax as monotherapy exhibits limited CNS penetration (33); nevertheless, synergistic antitumor activity can be obtained when it is combined with methotrexate, vincristine, and dexamethasone, resulting in superior overall efficacy. Rituximab was incorporated into the regimen based on immunohistochemical findings. Following this course of chemotherapy, the patient attained deep remission, providing a solid prerequisite for subsequent transplantation.

Multiple clinical trials, including SORMAIN (34) and MORPHO (35), confirm that post-transplant FLT3 inhibitor maintenance reduces relapse risk in FLT3-ITD-positive AML. However, our patient had MPAL with concurrent KIT D816V and FLT3-ITD. Per 2022 European LeukemiaNet (ELN) criteria, FLT3-ITD in the setting of wild-type NPM1 defines intermediate-risk disease. KIT D816V represents the major pathogenic driver of systemic mastocytosis, and acute leukemia associated with systemic mastocytosis carries a dismal prognosis. The patient’s bone marrow smear showed increased mast cell proportions; although systemic mastocytosis diagnostic criteria were unfulfilled, a KIT D816V-driven mast cell clone could not be ruled out. Concurrent KIT D816V and FLT3-ITD mutations (36) confer very poor outcomes that are not fully abrogated by allo-HSCT, underscoring the clinical need for dual-targeted maintenance. Conventional FLT3 inhibitors are type 2 kinase inhibitors binding to the inactive DFG-out conformation and lack activity against constitutively activated KIT D816V. By contrast, avapritinib (37, 38), a type I inhibitor targeting the active DFG-in conformation, inhibits both KIT D816V and FLT3-ITD, which provided the theoretical rationale for our regimen. The patient received avapritinib combined with pre-transplant chemotherapy. Sorafenib was started on post-transplant day +45 but discontinued because of grade 4 thrombocytopenia. Given the low baseline FLT3-ITD mutational burden and pre-transplant clearance of this mutation, we switched to single-agent avapritinib maintenance with serial NGS surveillance. Post-transplant NGS consistently showed FLT3-ITD and KIT D816V below the limit of detection, consistent with effective leukemic-clone eradication. This favorable outcome may stem from avapritinib’s dual-targeted activity together with the graft-versus-leukemia effect induced by allo-HSCT. Prospective studies are needed to establish optimal targeted-maintenance approaches for this high-risk subgroup.

TBI represents a classic myeloablative-conditioning platform regarded as first-line therapy for relapsed/refractory leukemia, especially in patients with extramedullary involvement. Ionizing radiation penetrates the BNB and bone cortex to directly clear leukemic blasts surrounding cauda equina nerve roots and eradicate extramedullary residual lesions unreachable by conventional chemotherapy. Furthermore, TBI is non-cross-resistant to most chemotherapeutic agents, generating synergistic complementary effects that reduce post-transplant relapse risk (10, 39, 40). A European retrospective analysis (41) reported superior outcomes with TBI-based myeloablative conditioning: 5-year overall survival 67.9% versus 55.5% (P = 0.0002) and cumulative incidence of relapse 37.5% versus 53.1% (P < 0.0001), relative to chemotherapy-only conditioning; nevertheless, radiation-associated organ toxicity remains a major drawback. fTBI mitigates radiation-related injury to normal tissues (e.g., lungs and peripheral nerves) via dose fractionation while preserving anti-leukemic potency, which constitutes its major advantage over single-dose TBI (10, 42, 43). For this patient, fTBI (5 Gy × 2 days; total dose 10 Gy) with 40% lung shielding was delivered. This strategy fulfilled therapeutic objectives without inducing radiation pneumonitis or neurotoxicity.

In the absence of a matched related donor, a fully matched unrelated donor was enrolled. We adopted an innovative triple stem cell infusion regimen (peripheral blood stem cell (PBSC) + UCBs + MSCs). This multicellular strategy synergistically accelerates hematopoietic reconstitution, mitigates GVHD, and potentiates the GVL effect (12, 44, 45). UCBs exhibit low immunogenicity and robust GVL activity, which collectively lowers GVHD risk in unrelated donor transplantation (11, 44). Moreover, UCB-derived natural killer and cytotoxic T cells exert potent cytotoxicity against chemotherapy-resistant and extramedullary leukemic residues (46). This immune clearance synergizes with fTBI to eradicate minimal residual disease within the cauda equina region. MSCs attenuate excessive allogeneic immune activation via immunomodulatory properties, further reducing GVHD incidence (47, 48). In addition, MSCs secrete neurotrophic factors, growth factors, and cytokines to facilitate hematopoietic engraftment and prevent delayed hematopoietic recovery (49, 50). These paracrine activities also promote cauda equina nerve repair and neurological functional restoration, which may account for the post-transplant improvement in the patient’s lower limb muscle strength. Multiple clinical studies support the safety and efficacy of UCB–MSC co-infusion. A multicenter retrospective study (45) reported a grade III–IV acute GVHD rate as low as 5% after combined UCB–MSC-supported HSCT. With a 76-month median follow-up, only one of 20 patients died of severe pulmonary infection, and no disease relapse was observed. Another multicenter trial (51) demonstrated that early post-transplant MSC infusion significantly reduced acute GVHD (17.7% vs. 47.9%, P < 0.001) and chronic GVHD (5.5% vs. 14.8%, P = 0.033) without increasing relapse risk.

Accumulated evidence confirms that UCB–MSC co-infusion ameliorates GVHD and improves survival in haploidentical or mismatched HSCT, while relevant data in matched unrelated donor settings remain limited. Our patient achieved rapid hematopoietic engraftment and experienced no severe GVHD after triple stem cell transplantation. Nevertheless, given the absence of a control group and mechanistic validation in this case report, and considering that matched unrelated donor HSCT alone can yield comparable favorable outcomes, a definite causal relationship between UCB–MSC co-infusion and the patient’s favorable prognosis cannot be established. Following upfront chemotherapy, PET/CT (**Figure 1B**) detected markedly diminished fluorodeoxyglucose (FDG) avidity in extramedullary lesions, whereas contrast-enhanced spinal MRI (**Figure 1D**) still displayed thickened, abnormally enhanced epidural soft tissue and cauda equina nerve roots, a metabolic–structural dissociation frequently observed in nervous system tumors. Such discrepancy stems from rapid leukemic blast metabolic suppression, contrasted with slow resolution of treatment-triggered inflammation, edema, and fibrotic tissue repair (52). Negative cerebrospinal fluid (CSF) cytology and progressive lesion regression on serial MRI verified that these imaging alterations reflected post-therapeutic changes rather than residual active disease.

This case represents the first documented MPAL with CES as the initial presentation, successfully treated with blood–nerve barrier-penetrating chemotherapy, fTBI myeloablation, and triple-source stem cell co-infusion. While nearly all previously reported similar cases ended in disease relapse or death, the present patient has achieved durable remission, partial neurological recovery, and long-term survival. This experience offers a novel therapeutic reference for managing such high-risk patients. Given the dismal intrinsic prognosis of MPAL with CES, clinicians should carefully balance the potential risks of this strategy—including graft failure, infection transmission, ectopic tissue formation, and high medical costs—against survival benefits. Strict patient selection and standardized institutional protocols are mandatory for safely implementing this innovative regimen.

As a single-case study, the present investigation has several inherent limitations. First, the independent contributions of UCB and MSCs cannot be definitively distinguished. Second, original pathological imaging slides were unavailable for retrospective review. Third, lumbar puncture and CSF analysis were not performed at initial diagnosis due to relative contraindications, including bilateral lower limb paralysis and multiple space-occupying lesions within the intervertebral foramina. Finally, although radiographic responses were favorable, we lacked CSF pharmacokinetic data to directly verify the CNS penetration efficiency of our treatment regimen.

## 4 Conclusion

MPAL with CES as the initial manifestation represents a rare clinical entity with dismal prognosis. The present case illustrates that an evidence-based multi-agent chemotherapy strategy followed by allo-HSCT supplemented with third-party UCB and MSCs may achieve successful salvage in this high-risk patient population.

## Patient perspective

The patient has shown significant clinical improvement, with partial recovery of activities of daily living, despite incomplete recovery of lower limb muscle strength.

## Data Availability

The raw data supporting the conclusions of this article will be made available by the authors, without undue reservation.
